# Towards Correlative Raman Spectroscopy–STEM Investigations Performed on a Magnesium–Silver Alloy FIB Lamella

**DOI:** 10.3390/nano15060430

**Published:** 2025-03-11

**Authors:** Jan Reimers, Martin Mikulics, Marta Lipinska-Chwalek, Berit Zeller-Plumhoff, Lidia Kibkalo, Maximilian Kruth, Regine Willumeit-Römer, Joachim Mayer, Hilde Helen Hardtdegen

**Affiliations:** 1 Ernst Ruska-Centre, Forschungszentrum Jülich, 52425 Jülich, Germany; j.reimers@fz-juelich.de (J.R.); m.lipinska@fz-juelich.de (M.L.-C.); l.kibkalo@fz-juelich.de (L.K.); m.kruth@fz-juelich.de (M.K.); j.mayer@fz-juelich.de (J.M.); 2 Institut für Metallische Biomaterialien, Helmholtz-Zentrum Hereon GmbH, Max-Planck Str. 1, 21502 Geesthacht, Germany; berit.zeller-plumhoff@hereon.de (B.Z.-P.); regine.willumeit@hereon.de (R.W.-R.); 3 Data-Driven Analysis and Design of Materials, Fakultät für Maschinenbau und Schiffstechnik, Universität Rostock, Albert-Einstein-Straße 2, 18059 Rostock, Germany; 4 Central Facility for Electron Microscopy (GFE), RWTH Aachen University, 52074 Aachen, Germany

**Keywords:** Mg-Ag alloys, carbon, correlative characterization, Raman spectroscopy, scanning transmission electron microscopy

## Abstract

In this study, a lamella prepared using focused ion beam (FIB) milling from a magnesium–silver alloy wire was investigated. The wire, intended for biomedical applications, was initially degraded in simulated body fluid (SBF) under physiological conditions. Raman spectroscopy was performed across the entire FIB specimen and the results were correlated with findings from scanning transmission electron microscopy (STEM). Our micro-Raman analysis identified chemical compounds at distinct regions within the specimen. Dominant Raman modes at ~1350 cm^−1^ and ~1590 cm^−1^, likely derived from elemental carbon from the FIB protection layer, were observed. Additionally, modes indicative of the alloy’s interaction with SBF, attributable to the constituents of SBF, were detected. Notably, Raman modes at ~3650 cm^−1^ corresponding to the OH stretching mode were identified in the targeted areas of the lamella, highlighting the chemical interaction between magnesium (Mg) and the SBF. The micro-Raman mapping images showed localized Mg(OH)_2_ distributions, which correlated strongly with the STEM analyses. This study underscores the effectiveness of correlating Raman spectroscopy, revealing chemical changes and STEM, capturing the corresponding microstructural changes. The combined approach is crucial for a deeper understanding of material degradation and reactivity in biocompatible alloys under physiological conditions and advances the characterization of biocompatible materials in physiological environments.

## 1. Introduction

The development of novel biocompatible materials is accompanied by efforts to achieve deep insights into physical, chemical, and biological processes down to the micrometer scale [1,2,3,4]. Among these materials, magnesium (Mg)-based alloys have gained significant attention for biomedical applications due to their unique combination of biocompatibility, biodegradability, and mechanical properties. However, their rapid degradation in physiological environments remains a major challenge [5,6]. To address this issue, recent studies have highlighted the potential of alloying Mg with elements such as silver (Ag) to enhance corrosion resistance and provide antibacterial properties, addressing critical issues in implant applications, such as postoperative infections and premature material failure [7,8,9]. Particularly promising in this context are Mg-based alloys in wire form, which have potential applications in biomedical devices such as sutures, stents, and guidewires. For these applications, achieving a balance between controlled degradation and mechanical performance is critical [10,11]. Despite the progress in material design, the precise mechanism governing Mg alloy degradation and their implications for biomedical performance require further investigation.

The degradation of Mg-Ag alloys in simulated body fluid (SBF) is primarily driven by electrochemical reactions, leading to the formation of various corrosion products. The process begins with the anodic dissolution of magnesium, accompanied by the cathodic reduction of water:

$$Mg\to {\mathrm{Mg}}^{2+}+2e^{-}$$

$${2H}_{2}O+2e^{-}\to H_{2}+2{OH}^{-}$$

As a consequence, the local environment becomes increasingly alkaline due to the accumulation of hydroxide (OH^−^) ions, resulting in the precipitation of magnesium hydroxide:

$${\mathrm{Mg}}^{2+}+2{\mathrm{OH}}^{-}\to \mathrm{Mg}{\left(\mathrm{OH}\right)}_{2}$$

Initially, this Mg(OH)_2_ layer provides temporary protection against further degradation. However, under physiological conditions, its stability is compromised by the presence of chloride ions (Cl^−^), which facilitate its dissolution and expose the underlying Mg substrate to continued degradation. As this protective layer degrades, the increasing local alkalinity promotes the nucleation and deposition of additional degradation products, including carbonates and calcium/phosphate compounds [2,6,12]:

$${\mathrm{Ca}}^{2+}+{{\mathrm{CO}}_{3}}^{2-}\to {\mathrm{CaCO}}_{3}$$

$${\mathrm{Mg}}^{2+}+{{\mathrm{CO}}_{3}}^{2-}\to {\mathrm{MgCO}}_{3}$$

$$3{\mathrm{Mg}}^{2+}+2{{\mathrm{PO}}_{4}}^{3-}\to {\mathrm{Mg}}_{3}{\left(PO_{4}\right)}_{2}$$

$$10{\mathrm{Ca}}^{2+}+6{{\mathrm{PO}}_{4}}^{3-}+2{\mathrm{OH}}^{-}\to {\mathrm{Ca}}_{10}{\left({\mathrm{PO}}_{4}\right)}_{6}{\left(\mathrm{OH}\right)}_{2}$$

The continuous exchange of medium under flow conditions prevents local saturation of Mg^2+^ ions, inhibiting the formation of a stable passivating layer and promoting sustained material loss. Moreover, localized corrosion phenomena—such as pitting and micro-galvanic effects—can emerge due to secondary phases or impurities within the alloy microstructure [13,14].

While advanced electron microscopy techniques provide detailed information on material composition and structure at the atomic scale [15,16], there is a compelling need for additional characterization methods that can clarify chemical bonding and compound identification. Ideally, these techniques should complement one another, offering comprehensive insights into the products of chemical reactions under controlled conditions. In addition, it is essential to develop a containment/bioreactor that accurately allows complex reactions to be performed in vitro under physiological conditions, enabling the simulation and observation of degradation mechanisms [17].

Traditional electron microscopy techniques are often limited in their ability to determine the chemical composition of materials, particularly in the context of dynamic chemical processes. In contrast, Raman spectroscopy, with its high chemical sensitivity—particularly towards elements like carbon—is ideally suited for studying aging, degradation, and corrosive mechanisms. In recent years, the integration of scanning electron microscopes with Raman spectroscopy has become commercially available [18,19]. However, the incorporation of (scanning) transmission electron microscopy with Raman spectroscopy within a single chamber, although reported sporadically [20,21,22,23], remains commercially unavailable. Challenges persist, particularly with the precise positioning and focusing of a laser beam for Raman analysis at the specific location of the transmission electron microscopy (TEM) analysis and effectively collecting the scattered light from the specimens. Given these technical hurdles, the characterization of TEM specimens, specifically FIB-prepared lamellae, using Raman spectroscopy remains a challenge in in situ TEM techniques. Consequently, employing a correlative approach that combines both characterization techniques in an ex situ setting is not only necessary, but also the core methodology of our study. Unlike bulk-scale scanning electron microscopy/energy dispersive X-ray spectroscopy (SEM/EDX), which provides large-area compositional data, our correlative method ensures that chemical and structural transformations are mapped at the exact same location at the nanoscale. This direct correlation between Raman spectroscopy and scanning transmission electron microscopy-energy dispersive X-ray spectroscopy (STEM-EDX) allows for a more precise understanding of localized degradation mechanisms, revealing how microstructural features influence chemical transformations in different regions of the degradation layer. This work focuses on the characterization of a lamella prepared by the FIB technique, aiming to bridge the gap between microstructural insights and chemical interactions while maintaining a high level of spatial correlation.

## 2. Materials and Methods

A wire with a diameter of 80 µm was prepared from a magnesium–silver alloy containing 4 weight percent of silver (Mg-4Ag). The fabrication procedure involved melting pure Mg (99.98% purity, MAGONTEC, Sydney, Australia) and alloying it with Ag (99.99% purity, ESG Edelmetall-Service GmbH & Co. KG, Rheinstetten, Germany) under a protective argon + 3 vol% SF_6_ atmosphere to prevent oxidation. The melt was cast into a billet using a modified permanent direct chill casting technique and was subsequently homogenized at 450 °C for 16 h to achieve a uniform composition. The homogenized billet was then hot-extruded at 425 °C with a mean exit speed of 3.1 m/min and an extrusion ratio of 1:625, producing 1 mm diameter Mg-4Ag wires. These wires were then drawn down to a final diameter of 80 µm using the “EZ2.01” drawing machine (Müller Engineering GmbH Co. KG, Todtenweis, Germany). To facilitate the drawing process, drawing wax was applied until the wire diameter reached 320 µm. Each drawing pass reduced the wire cross-sectional area by approximately 20%, with a drawing speed of 0.25 m/s. To prevent embrittlement and reduce work hardening, the wires underwent recrystallization annealing at 425 °C for 5 min after every second drawing pass. Based on prior studies using identical material and fabrication procedures, the microstructure of the Mg-4Ag alloy was expected to consist of a fine-grained α-matrix with grain sizes in the range of 35–45 µm. Additionally, no intermetallic precipitates were anticipated after heat treatment, as previously reported [17,24]. The wire specimen was then subjected to simulated body fluid [25] under physiological conditions that mimic those in which the alloy is intended for future application. For this purpose, a bioreactor equipped with a flow cell was used, allowing precise control of key parameters. The degradation experiment was carried out for approximately 6 h. The temperature was maintained at 37 °C, and the pH was adjusted to 7.4 using CO_2_ gas. Additionally, the flow rate was set to 1 mL/min [17]. The SBF, besides hydrochloric acid, contained sodium and calcium chloride, sodium bicarbonate (NaHCO_3_), and phosphate (Na_2_HPO_4_).

Following the degradation experiments, electron microscopy investigations were carried out to further investigate the microstructural changes. To this end, a ~100 nm thin FIB lamella was prepared using the Dual-FIB workstation FEI Helios NanoLab 400S (Thermo Fisher Scientific Inc., Waltham, MA, USA). The wire surface was coated with a 3 μm carbon protective layer using both electron and Ga ion beam deposition. Phenanthrene was used as the carbon precursor. The first layer was deposited using an electron beam (5 kV, 11 nA), while the second layer was deposited using a Ga ion beam (30 kV, 0.28 nA). Cross-sectional trenches were milled above and below the regions of interest with an ion beam (Ga, 30 kV and 5.5 nA). The lamella was then transferred to a copper grid using a micromanipulator needle after edge cleaning and undercutting. Further thinning and polishing were performed using the accelerating voltage (5–30 keV) and beam current (2.8 nA–48 pA). For structural and chemical analysis, high-angle annular dark-field scanning transmission electron microscopy imaging (HAADF STEM) and energy-dispersive X-ray analysis (EDX) were conducted with a probe-corrected FEI Titan G2 80–200 operated at 200 kV [26] using Velox Software (Thermo Fisher Scientific Inc., Waltham, MA, USA). The HAADF detector was operated with inner and outer semi-collection angles of 69–200 mrad using a camera length of 110 mm. The microscope was operated with a semi-convergence angle of 24.7 mrad and a spot size of 6 nm. The EDX maps were acquired with a scan pixel size of 4.129 nm and a dwell time of 10 µs, ensuring a high spatial resolution in chemical analysis. Further details can be found in [17].

Raman studies were conducted on the lamella using a micro-Raman microscope in backscattering geometry (Renishaw inVia FSM REFLEX, New Mills, Gloucestershire, UK). A frequency-doubled Nd-YAG laser (532 nm, 50 mW) was employed as the excitation source, and a CCD array detector was used to capture the signals. A 100-fold objective lens (NA 0.85) was chosen and the laser spot size was ~1 µm. The spectrometer was calibrated to the transverse optical phonon of Si at 521 cm^−1^. Spectra were recorded over the range of 100 cm^−1^ to 4000 cm^−1^. Great care was taken not to destroy the lamella and, therefore, the laser power was kept below 0.1 mW to avoid sample damage and any heating effects [27].

## 3. Results

### 3.1. Microstructural Analysis Using HAADF STEM and EDX

**Figure 1:**
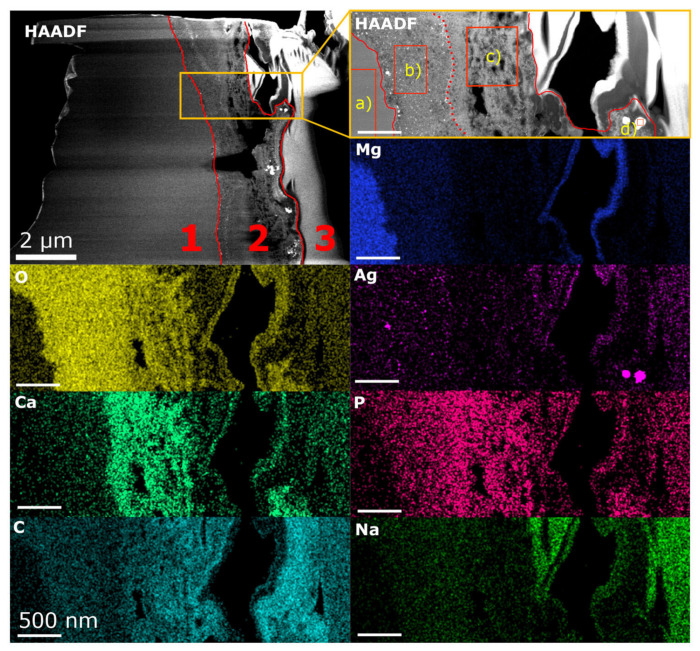
HAADF overview image of the Mg-4Ag lamella after degradation in SBF, revealing three distinct regions: (1) bulk (non-degraded) material, (2) the degradation layer, and (3) the FIB protection layer. Elemental maps are shown as weight percentage data, and elemental composition analyses were conducted within the highlighted orange rectangle at four different regions of interest: a), the bulk area, b), the inner degradation layer with fine pores, c) the outer sub-layer with irregularly distributed pores, and d) unevenly distributed light areas. The figure is reprinted from *ACS Appl. Mater. Interfaces* 2023, 15, 29, 35600–35610 [17]. Copyright 2023 American Chemical Society, Creative Commons Attribution 4.0 License (CC BY 4.0, https://creativecommons.org/licenses/by/4.0/).

Figure 1 shows a high-angle annular dark field (HAADF) overview image of the TEM lamella under investigation. Three distinct regions, 1–3, are discernible, related to the bulk Mg-4Ag alloy, the degradation layer, and the FIB protection layer, respectively. The regions of interest (ROIs) are outlined by an orange rectangle where EDX studies were performed, with further details provided in an inset on the right-hand side, along with corresponding elemental distribution maps, below. The average composition values of the individual ROIs, indicated as a)−d) in the HAADF inset of Figure 1, were determined. Region a) consists of non-degraded bulk material exhibiting a homogeneous composition, likely due to the annealing of the solid-solution Mg alloy, with no precipitates visible in the HAADF images. Region b), an inner degradation layer located adjacent to the bulk material, features fine, evenly distributed pores and precipitates (or agglomerates) of Ag. Region c), an outer sub-layer, contains large, irregularly distributed pores, as well as Ag-enriched nanoprecipitates/nanoagglomerates (significantly smaller and less populated, as in region b)). Region d) reveals unevenly distributed bright areas that correspond to Ag-enriched zones. The microstructural differences between the two degradation layers differ not only in morphology, but also in chemical composition, affecting the concentrations of Mg, O, and Ca. The quantitative EDX analysis indicates that the inner degradation layer primarily exhibits high levels of Mg and O, with lower concentrations of Na, P, Cl and Ca, while the outer layer shows a decrease in Mg and O content, with a simultaneous increase in Ca and P.

### 3.2. Raman Spectroscopy

**Figure 2:**
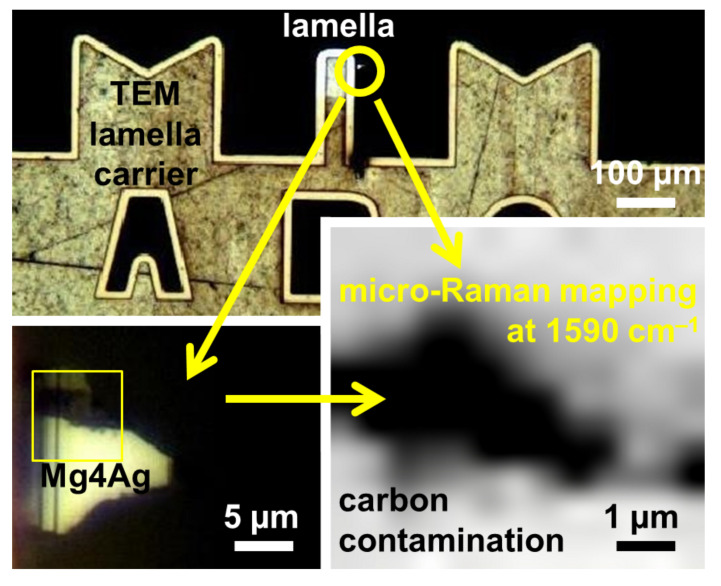
Optical micrograph of a TEM lamella carrier of a Mg-Ag alloy (top), detailed microscope image of the investigated sample (bottom left), where the area of the micro-Raman mapping is marked by a yellow rectangle, and micro-Raman mapping at 1590 cm^−1^ from the Mg-4Ag alloy lamella (bottom right), corresponding to the Raman mode attributed to elemental carbon. The Raman mode intensity is depicted graphically using a grayscale color scheme (black–white).

Figure 2 presents an optical micrograph of the TEM lamella carrier alongside the Mg-4Ag lamella studied with Raman spectroscopy, showing an overview (top) and a detailed view of the FIB lamella area (bottom left). The corresponding Raman intensity mapping at 1590 cm^−1^, attributed to elemental carbon, is shown in grayscale in the bottom right. This mode’s intensity is primarily observed near the outer rim of the lamella, consistent with the FIB milling process, where carbon was applied for protection. Representative Raman spectra from the examined region of the FIB lamella, highlighting both the 1350 cm^−1^ and 1590 cm^−1^ modes associated with the elemental carbon, are displayed in Figure 6a (blue). Notably, the Raman intensity is highest near the outer rim and decreases significantly in the deeper regions of the lamella. A similar intensity distribution for both modes near the lamella’s outer rim was observed, but it is not shown here.

**Figure 3:**
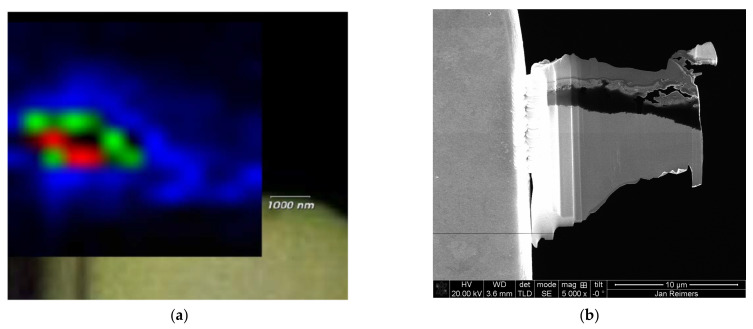
(**a**) Micro-Raman intensity mapping image displaying measurements at the Raman mode 3650 cm^−1^, collected from the target region of interest on the Mg-4Ag lamella. The intensity is presented in colors: from blue (low intensity), through green (intermediate intensity), to red (high intensity). The lowest intensity is at the outer rim of the lamella and in the non-degraded Mg-4Ag area. (**b**) Detailed SEM image of the investigated lamella. The top of the lamella is non-degraded/compact related to the carbon protective layer, followed by the SBF-degraded region (non-uniform in structure, pores of different sizes) and the non-degraded Mg-4Ag area.

Having established the presence of elemental carbon through spectroscopy, it was anticipated that the Mg-4Ag alloy would undergo notable reactions with the SBF in the bioreactor. A likely indication of these reactions is the dissolution of the alloy, evidenced by the formation of Mg(OH)_2_, which can be identified by the emergence of the Raman (OH) stretching mode at ~3650 cm^−1^ [28,29]. Indeed, this mode was observed in the SBF-degraded region of the lamella specimen, as shown in the mapping presented in Figure 3a. The SEM image in Figure 3b provides a detailed view of the lamella. The variations in intensity observed in the mapping suggest the possibility of different concentrations of the formed compound.

**Figure 4:**
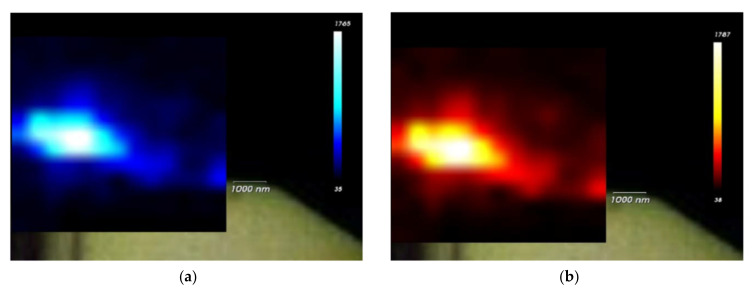
Micro-Raman mapping of the lamella structure. Graphical presentation of Raman modes in color. (**a**) Mapping at 1400 cm^−1^ (blue) and (**b**) at 1450 cm^−1^ (red). Both mappings highlight Raman modes attributed to the asymmetric stretching of carbonate groups.

**Figure 5:**
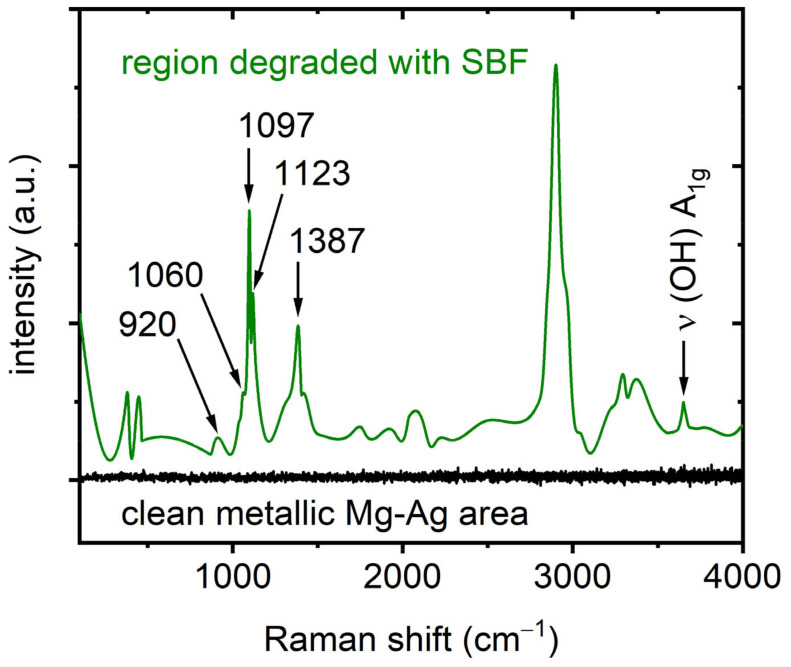
Representative Raman spectrum collected from the “degraded” region from the lamella where the (OH) stretching mode was detected (green/olive color). The scanning parameter for the presented spectrum (green/olive color) was taken as 10 s, 10 scans were accumulated, and the adaptive smoothing algorithm (Raman spectrometer software—Renishaw WiRE 5) was applied. For the sake of comparison, the Raman measurements (scanning parameter 10 s and accumulation only once) were performed on the clean “metallic” area (to exclude any presence of oxides, etc.), and they are presented in black. The microscope image of the investigated lamella is presented in Figure 2. Please note that the investigated lamella`s area was contaminated (during lamella fabrication) with carbon-related compounds (discussed later in this text). Raman modes at ~ 1060 cm^−1^ [34,35], 1097 cm^−1^ [36,37], and 1123 cm^−1^ [30], as well as ~920 cm^−1^ [38], are related to different carbonates and phosphates.

Building on these observations, further micro-Raman mapping across the lamella’s region of interest uncovers the formation of additional chemical products post-reaction. For example, the formation of carbonates is observable by mapping the intensity of selected Raman modes at 1400 cm^−1^ and at 1450 cm^−1^, as presented in Figure 4a and Figure 4b, as color-coded intensity images. These modes are attributed to the asymmetric (CO) stretching mode of the carbonate group [30]. Although their respective micro-Raman mapping intensities collected over a large area varied strongly (up to a factor of 100), the highest intensities were detected in identical lamella regions, as is visible when comparing both images. Furthermore, in this region, intense Raman modes around 1100 cm^−1^ and ~1120 cm^−1^ are observable due to the symmetric (CO) stretching mode of the carbonate group [30]. They are visible in Figure 5 (green trace) and Figure 7.

**Figure 6:**
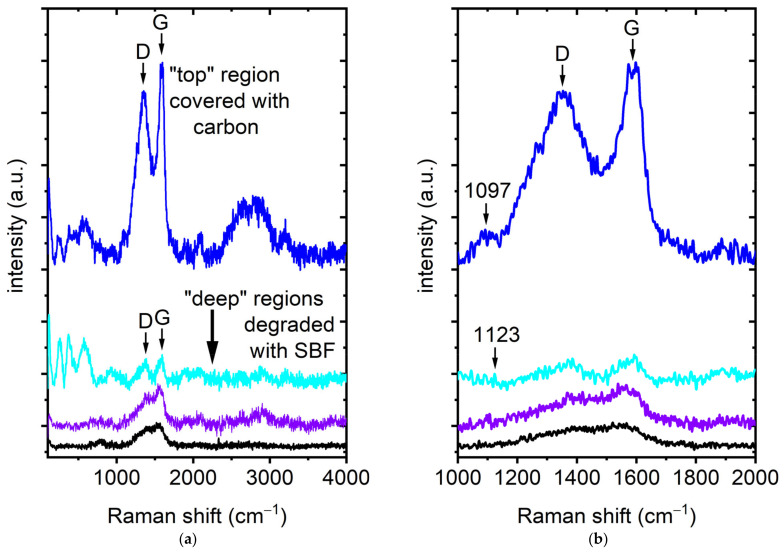
(**a**) Raman spectra showcasing the “top” region coated with carbon alongside deeper regions impacted by SBF degradation; (**b**) presents these spectra with enhanced detail. Notably, the D and G modes of elemental carbon are observable, along with distinct modes at ~1097 cm^−1^ and ~1123 cm^−1^, attributed to the ν_1_ CO_3_^2−^ symmetric stretching modes of CaCO_3_ [36,37] and MgCO_3_ [30], respectively.

**Figure 7:**
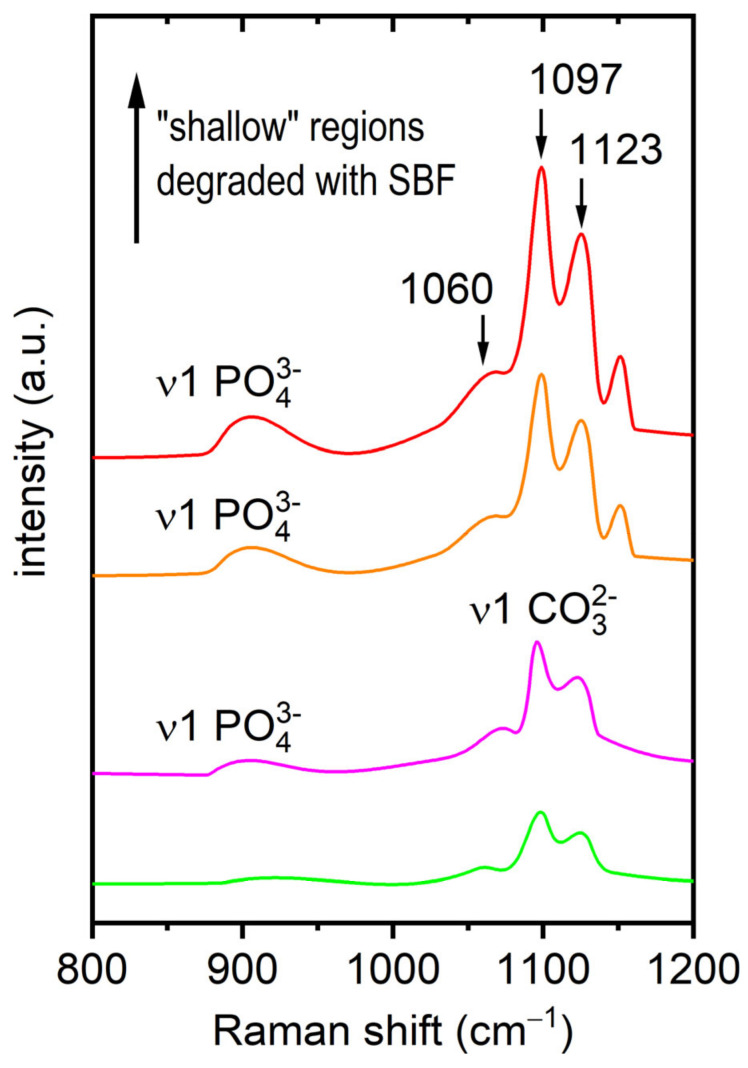
Representative Raman spectra from “shallow” regions affected by SBF. The scanning parameter for the presented spectra was taken as 10 s, 10 scans were accumulated, and the adaptive smoothing algorithm (Raman spectrometer software—Renishaw WiRE 5) was applied. Distinct ν_1_ CO_3_^2−^ symmetric stretching modes are observable: sodium bicarbonate at ~1060 cm^−1^, [34,35] CaCO_3_ at ~1097 cm^−1^ [35,36], and MgCO_3_ at ~1123 cm^−1^ [30], as well as the ν_1_ PO_4_^3−^ symmetric stretching mode at ~920 cm^−1^ [38].

Following the analysis of the carbonate formations, additional insights were revealed through Raman spectroscopy of various regions of the FIB lamella. Figure 5 displays a representative Raman spectrum from areas where the (OH) stretching mode is observed (target area, depicted in green/olive). The scanning parameter for the presented spectrum (green/olive color) was taken as 10 s, 10 scans were accumulated, and the adaptive smoothing algorithm (Raman spectrometer’s software—Renishaw WiRE 5) was applied. Modes were discerned at ~1060 cm^−1^, 1097 cm^−1^, and 1123 cm^−1^, as well as ~920 cm^−1^, and these will be discussed in more detail later in this study. They are related to different carbonates and phosphates (see also Figure 7). Furthermore, modes were also observed in the range between ~1387 cm^−1^ and ~3080 cm^−1^, the range in which (CH) and (CC) stretching vibrations of different hydrocarbons [31,32] are observed. Here, it should be noted that in Figure 5, the typical signatures for the D and G bands attributed to the amorphous/graphitic carbon of the protective carbon layer were not unambiguously observed. The origin of the differing Raman signatures of carbonaceous compounds/structures visible in Figure 5, as well as in Figure 6, can be attributed to the non-homogeneously distributed carbonaceous compounds/structures across the lamella as a consequence of the preparation procedure (using an electron beam and a Ga ion beam, as described in the Materials and Methods section). The carbon precursor, phenanthrene—used to deposit carbon as the protective layer on the degraded material in the FIB—and its preparation procedure may be responsible for the modes observed. We assume that the decomposition of the precursor was not complete. Therefore, a signature related to phenanthrane [32] and/or its decomposition products, such as different hydrocarbons [31], may also be detected. Furthermore, it cannot be excluded that ion-induced defects [33] in the “carbon” protection layer will additionally affect the intensity of the D and G modes of amorphous and graphitic carbon. The compounds, however, do not emerge in the degradation process of the alloy itself and are not dealt with in this report. They will be the focus of our future comprehensive study. In contrast, in the deeper regions, where the bulk material resides, and which are non-degraded, the signal is only noise (depicted in black) and lacks any Raman signatures indicative of a clean metal (Mg-4Ag) surface, from which no Raman modes are typically expected. In contrast, a distinct signature is observable in areas closer to the “surface” of the wire, i.e., the rim of the lamella, due to the exposure of the wire to the SBF.

The objective is to identify the chemical compounds from the fingerprints observed in the spectra measured and, thereby, to gather more detailed chemical information about these compounds and their reaction pathways. Representative spectra, recorded in different regions of the lamella, are presented as follows: In Figure 6a, spectra collected near the top region of the lamella display signatures indicative of elemental carbon. In deeper regions, the intensity of the Raman mode is lower, but still detectable, indicating that the material remains predominantly metallic with minimal degradation, although some degradation products are observable. Detailed spectra are shown in Figure 6b. Near the top region, a mode at ~1097 cm^−1^ becomes discernible, attributed to the ν_1_ CO_3_^2−^ symmetric stretching mode of CaCO_3_ [36,37] and another mode at ~1123 cm^−1^, attributed to MgCO_3_ [30].

In Figure 7, the spectra from the shallower regions display distinct modes attributed to the degraded alloy: a mode at ~920 cm^−1^, which may be attributed to the ν_1_ PO_4_^3−^ symmetric stretching mode [38], is observable, as well as the ν_1_ CO_3_^2−^ symmetric stretching mode of different carbonates at ~1060 cm^−1^ [34,35], along with further intense modes at ~1097 cm^−1^ [36,37] and ~1123 cm^−1^ [30].

## 4. Discussion

In general, the corrosion of Mg-4Ag alloys in SBF can be described by a series of electrochemical reactions. The anodic reaction involves the dissolution of magnesium, $Mg\to {Mg}^{2+}+{2e}^{-}$, while the cathodic reaction is primarily the reduction of water, ${2H}_{2}O+{2e}^{-}\to H_{2}+{2OH}^{-}$. The resulting ${Mg}^{2+}$ ions combine with ${OH}^{-}$ to form magnesium hydroxide (${Mg(OH)}_{2})$, which can further interact with ambient ${CO}_{2}$ or dissolved carbonates to produce magnesium carbonate (MgCO_3_). In parallel, calcium ions present in the SBF can lead to the formation of calcium carbonate (CaCO_3_), and phosphate ions may contribute to the precipitation of magnesium phosphate (Mg_3_(PO_4_)_2_ and hydroxyapatite (Ca_10_(PO_4_)_6_(OH)_2_. Mg is particularly reactive and forms a hydroxide; hence, the (OH) stretching mode is evident. These hydroxides are likely to react with ambient CO_2_, leading to carbonate formation. Additionally, constituents from the SBF may contribute to the observed spectra features. No Raman signals are expected from the chlorides of sodium or calcium (components in the SBF), as they are Raman inactive at ambient conditions. Therefore, our focus was directed toward spectral regions indicative of the carbonates and phosphates used in the SBF, such as sodium bicarbonate (NaHCO_3_) and disodium orthophosphate (Na_2_HPO_4_), with the most intense modes expected between ~900 cm^−1^ and ~1000 cm^−1^ [34,38]. As detailed in Figure 7, a mode at ~920 cm^−1^, which may be attributed to the ν_1_ PO_4_^3−^ symmetric stretching mode [38], was observed. Furthermore, the expected ν_1_ CO_3_^2−^ symmetric stretching mode of sodium bicarbonate at ~1060 cm^−1^ [34,35] was also detected, along with further intense modes at ~1097 cm^−1^ and ~1123 cm^−1^, which were again attributed to CaCO_3_ [35,36] and MgCO_3_ [30], respectively.

The correlation between the Raman spectroscopy results and the microstructural analysis of the Mg-4Ag lamella was influenced by several challenges during the sample preparation and transfer. The inherent fragility of the lamella, particularly after thinning to approximately 100 nm using the FIB technique, significantly increased the risk of mechanical damage. This fragility is problematic for Mg-4Ag alloys, which exhibit not only proneness to corrosion and structural degradation, but also pores and cracks in the degradation layer. The thinning process was particularly challenging due to the significant difference in mechanical properties between the bulk Mg-4Ag material, which is soft and prone to deformation, and the harder, more brittle degradation layer, leading to uneven material removal and significant curtaining effects. During the FIB milling and subsequent transfer, part of the lamella broke off, as shown in Figure 3b. This breakage raises concerns about how mechanical disruption might affect the overall analysis. Such disruptions may affect the interpretation of results, particularly by introducing artifacts or altering the observed microstructure. To mitigate these issues, a protective carbon layer was applied prior to the FIB milling, and final polishing was carried out using reduced ion beam energies. These measures were implemented to minimize ion-induced damage and mechanical stress during the thinning process. Nevertheless, residual effects, such as curtaining and ion contamination, may influence the integrity of EDX maps and Raman spectroscopy data. These challenges emphasize the need for further optimization of FIB milling parameters and transfer techniques to reduce mechanical stress and ensure more reliable and consistent results in future studies.

Despite the challenges encountered during the sample preparation, the Raman spectroscopy provided valuable insights into the material’s condition. In particular, the analysis of deeper regions of the lamella revealed weak or undetectable Raman modes (Figure 6), suggesting either the presence of the pure alloy or slight partial degradation. In these regions, the absence of significant Raman signals or the detection of an emerging (OH) stretching mode at approximately 3650 cm^−1^ indicates a largely unreacted alloy core or initial stages of degradation. Additionally, low-intensity modes related to elemental carbon were observed at ~1350 cm^−1^ and ~1590 cm^−1^. These modes correspond to region (1) in the HAADF image and region a) in the EDX study, in which a homogeneous Mg-4Ag alloy with trace amounts of oxygen and carbon was identified.

Shifting to the shallower regions of the lamella, the Raman analysis revealed more distinct chemical activity. The detection of Raman modes at ~1060 cm^−1^ and ~920 cm^−1^, attributed to sodium bicarbonate (NaHCO_3_) [34,35] and disodium orthophosphate (Na_2_HPO_4_) [38], respectively, suggests the incorporation of SBF constituents into the degradation layer. This highlights how the chemical environment in these regions was significantly altered by interactions with the SBF, leading to the formation of carbonate and phosphate compounds. Moreover, the presence of intense and narrow Raman modes at ~1097 cm^−1^ and ~1123 cm^−1^, attributed to the ν_1_ CO_3_²^−^ symmetric stretching modes of CaCO_3_ [35,36] and MgCO_3_ [30], respectively, indicates that carbonate compounds formed as a result of the alloy’s interaction with the SBF. The shift in the chemical composition of the degradation layer—marked by the formation of calcium and magnesium carbonates—signals a progression in the corrosion process, driven by prolonged SBF exposure. Such findings align with previously reported studies [39], which similarly identified carbonate formations in magnesium-based alloys under comparable conditions. Additionally, these results are supported by the HAADF and EDX analyses, which show a decrease in magnesium and oxygen levels, accompanied by an increase in calcium and phosphorus concentrations from the inner to the outer sublayers of the degradation region (regions (2), b), and c)).

While these findings are conclusive regarding carbonate formation, the potential presence of hydroxyapatite (HA) remains less certain. Although our Raman spectroscopy studies did not definitively confirm the formation of HA, the detection of phosphate groups in the shallow regions of the lamella suggests that HA formation might have been possible. The EDX analysis, which showed an increase in calcium and phosphorus, indicates that the conditions necessary for HA formation may have been present. Previous studies, such as [40,41], have shown that HA formation is closely linked to calcium and phosphate interactions under physiological conditions. However, in our study, the Raman spectra did not show the characteristic ν_1_ PO_4_³^−^ modes typically associated with crystalline HA. This may have been due to incomplete crystallization or the formation of amorphous or poorly crystalline HA, as observed in other studies ([39,42]). Alternatively, the experimental time constraints may not have allowed sufficient time for HA crystallization, or the rapid degradation of magnesium may have interfered with HA formation. In fact, faster magnesium degradation can promote the dissolution of magnesium ions, preventing the stable formation of carbonate and phosphate phases [39]. Thus, while the EDX data suggest the potential for HA formation, this cannot be conclusively confirmed without additional evidence from Raman spectroscopy or other complementary techniques. Our results are consistent with previous studies in which incomplete HA formation or amorphous phosphate phases were observed. Further investigations—particularly involving longer SBF exposure times or complementary techniques, such as X-ray diffraction (XRD)—could help to clarify the potential formation of HA in future studies.

Lastly, the comparison between the Raman spectroscopy results and the STEM analysis of the carbon-protected regions of the lamella revealed a clear dominance of the D and G bands, characteristic of elemental carbon. These Raman findings strongly correlate with the results from the HAADF imaging (region (3)) and EDX analysis (region d)), confirming that this layer is primarily composed of carbon, with minor amounts of oxygen, reduced magnesium (Mg), and increased silver (Ag) compared to the bulk alloy. This correlation between Raman spectroscopy and microstructural techniques discloses the potential of using such a correlative approach to gain comprehensive insights into the surface composition. The elemental carbon observed in the D and G bands is likely to have been due to the carbon coating applied during the FIB milling process to protect the lamella. The presence of oxygen in this region may suggest minor oxidation, potentially introduced during sample preparation or environmental exposure, or through interaction with the degradation medium. Moreover, the reduction in Mg and the increase in Ag, as identified by EDX, cannot be detected by Raman spectroscopy alone, as these metals are not Raman-active. This highlights the need for further correlative analysis to fully understand the surface composition and transformations.

While this correlative study between Raman spectroscopy and STEM on a FIB lamella demonstrates the ability to locally identify chemical compounds and relate them to the microstructure, further investigations are needed to fully understand the degradation mechanisms of Mg-Ag alloys. Our findings confirmed the formation of carbonate and phosphate compounds, such as MgCO_3_ and CaCO_3_, during the degradation process in the SBF, which is consistent with previous studies that highlight the role of environmental ions in driving corrosion. However, additional work is necessary to explore whether longer exposure times or different environmental conditions lead to the complete formation of hydroxyapatite (HA) or other relevant compounds. Although our study did not conclusively confirm HA formation, the presence of phosphate groups suggests that incomplete or amorphous HA may have formed, which has also been observed in other research. Future work should include the investigation of bulk materials at various stages, from initial sample fabrication to the final lamella positioning on a TEM grid, to better track the progression of degradation. Investigating both before and after microstructural analysis would help in identifying and excluding impurities introduced during the fabrication process, providing a more accurate understanding of the reaction pathways. Moreover, expanded Raman studies of the SBF and its constituents are recommended to fully leverage the correlative potential of Raman spectroscopy and STEM in identifying and characterizing the chemical products formed during degradation.

## 5. Conclusions

In this study, we performed correlative Raman spectroscopy and STEM investigations on a magnesium–silver alloy FIB lamella to gain deeper insights into the degradation process of Mg-based biomaterials. Our findings provide a localized understanding of degradation mechanisms by identifying chemical compounds and correlating them with microstructural features at the micrometer scale. This approach enhances the interpretation of corrosion processes in simulated physiological environments (in vitro). The results highlight the importance of correlative characterization techniques in advancing the development of biocompatible implants. By mapping chemical and structural changes at high resolution, this study supports strategies for optimizing alloy composition and surface treatments to improve implant longevity, degradation resistance, and biocompatibility. Specifically, the observed formation of carbonates (MgCO_3_, CaCO_3_) and phosphate-related compounds suggests a pathway for hydroxyapatite (HA) formation, a key factor in implant integration. However, the absence of clear HA formation within the experimental timeframe indicates that the degradation period may not have been sufficient to capture its nucleation and growth.

This limitation underscores the need for future studies to assess long-term degradation behavior and HA formation under extended exposure conditions. Furthermore, investigating the influence of biological factors, such as protein interactions and cellular activity, through cell culture and in vivo studies, will be essential for understanding the integration of Mg-based implants in physiological environments. Expanding correlative workflows into a multiscale approach by including techniques such as in situ synchrotron radiation-based nano-CT (nano-computed tomography) and SEM/EDX could enable a more comprehensive understanding of the degradation mechanisms of Mg-based alloys, ultimately advancing their suitability for biomedical applications.

## Data Availability

Experimental data are available upon reasonable request from the corresponding authors.
